# Clinical and Laboratory Predictors of Mortality in COVID-19 Infection: A Retrospective Observational Study in a Tertiary Care Hospital of Eastern India

**DOI:** 10.7759/cureus.17660

**Published:** 2021-09-02

**Authors:** Deependra Rai, Alok Ranjan, Ameet H, Sanjay Pandey

**Affiliations:** 1 Respiratory Medicine, All India Institute of Medical Sciences, Patna, Patna, IND; 2 Community and Family Medicine, All India Institute of Medical Sciences, Patna, Patna, IND; 3 Pulmonary Medicine, All India Institute of Medical Sciences, Patna, Patna, IND

**Keywords:** covid-19, mortality, covid-19 outcome predictor, inflammatory marker, medical comorbidities

## Abstract

Introduction

COVID-19 is associated with huge morbidity and mortality in India. Identification of factors associated with mortality would make a difference in the management of COVID-19 infection-related illness.

Objective

To assess clinical & laboratory parameters associated with adverse outcomes among 984 patients with COVID-19 infection admitted to a tertiary care hospital in eastern India.

Materials and methods

All patients with real-time polymerase chain reaction (RTPCR) or rapid antigen positive for COVID-19 admitted at our All India Institute of Medical Sciences (AIIMS) Patna between 1st July to 30th Aug 2020 were included for analysis. Statistical analysis was performed using Stata, version 10 (Stata Corp, College Station, USA). Four subgroup regression models have been analyzed to predict the odds of death.

Results

A total of 984 COVID-19 cases admitted to our hospital during the given period were analyzed. Out of 984 cases, 762 (77.44%) were males and 222 (22.56%) females. The overall case-fatality rate among admitted cases was 254 (25.81%) [males (26.64%) and females (22.96%)]. The final logistic regression model showed that patients presenting with severe COVID-19 disease (adjusted odds ratio [aOR]: 17.81), cough (aOR: 3.83), dyspnea (aOR:2.35), age 60-75 (aOR:1.47), age >75 years (aOR:3.97), presence of chronic kidney disease (CKD) (aOR:2.95), were found to be significantly associated with a high risk of mortality after controlling for the confounders (p<0.05). Among lab variable, total leukocyte count (TLC) (>10,000/mm3) (aOR: 1.74), neutrophil-lymphocyte ratio (NLR) (>3.3) (aOR:2.37), serum albumin (<3.4 g/dl) (aOR : 2.3), blood urea (>43 gm/dl) (aOR:3.72), ferritin (>322) (aOR:2.39), and D-dimer (>0.5) (aOR:5.58) were significantly associated with higher mortality (p<0.05)

Conclusion

Age 60 years plus, presence of CKD, and severe covid infection carried the highest risk of mortality. Lab markers such as raised TLC, ferritin, D-dimer, and low albumin were associated with worse outcomes in our subset of COVID-19-related illness.

## Introduction

Coronavirus disease (COVID-19) is an infectious disease caused by the severe acute respiratory syndrome coronavirus 2 (SARS-CoV-2) [1]. India has reported 13 million cases, with more than 0.15 million people succumbing to the disease by 14th April 2021. [2] Studies have shown that 14% of COVID-19-associated pneumonia cases progress to become severe, and 5% of infected patients require intensive care [3]. Once the disease becomes severe, COVID-19 cases present with features of acute respiratory distress syndrome (ARDS), multi-organ dysfunction, and eventually death. The case fatality rate has varied from less than 1% in some countries to 18.3% in the Lombardy region of Italy [4].

India has unique socio-cultural differences compared to the Western world. Identification of clinical characteristics and risk factors that can potentially distinguish between high and low risk of mortality in an Indian population will lead to improved risk assessment and better clinical management. This study has been conducted with the aim to identify factors associated with a higher risk of mortality in COVID-19 patients needing admission and determine the case fatality rate of our institute which would help in early intervention and aggressive treatment.

## Materials and methods

This was a single-center, retrospective, medical record-based observational study involving all COVID-19 patients requiring admission at a tertiary care hospital All India Institute of Medical Sciences, Patna, India. **After the approval of the ethics committee**, records of all patients admitted to the institute between 1st July 2020 to 30th August 2020 were evaluated.

Data collection

Two post-graduate residents collected data in a predesigned proforma from patient records present in the medical record department which was reviewed and cross-checked for validation by two faculty members from pulmonary medicine and the department of community medicine. Retrieved data include demographic information, exposure history, clinical symptoms, comorbidities, and laboratory test results, details of treatment provided, length of hospitalization, complications, and outcome. The severity of covid infection was defined according to guidelines issued by the Ministry of Health and Family Welfare, Government of India (MoHFW) [5]. All patients admitted to our institute are investigated and treated as per the COVID-19 treatment protocol of our institute. A baseline battery of investigations was performed and based upon clinical characteristics and severity, patients are triaged. If fulfilling the admission criteria, severely ill patients are shifted to our intensive care units else they are shifted to various treatment wards. The following investigations are routinely done on admission. Hematological: complete blood counts, blood sugar levels, liver function test, kidney function test, prothrombin time, international normalized ratio (INR), D-dimer, and inflammatory markers like C-reactive protein (CRP), pro-calcitonin, and serum ferritin. Viral markers for hepatitis B and C and HIV are also noted. Chest X-ray, ECG, arterial blood gases, and glycosylated hemoglobin whenever indicated are ordered. 

Definitions of mild, moderate, and severe COVID-19 [5]

Mild: No evidence of breathlessness or hypoxia (normal saturation).

Moderate: Breathlessness and/or hypoxia (saturation 90-94% on room air), respiratory rate of 24 or more, and no features of severe disease.

Severe: Any of the following: severe respiratory distress, oxygen saturation < 90% on room air, respiratory rate > 30, or shock or evidence of life-threatening organ dysfunction.

Statistical analysis

All statistical analysis was performed using Stata, version 10 (Stata Corp, College Station, USA). Shapiro-Wilk test was used to assess the normality of continuous variables, especially the lab parameters. For non-normally distributed data, non-parametric tests such as Mann-Whitney and Wilcoxon Rank test were used to compare the equality of distribution of continuous variables, which were presented as median with interquartile range, otherwise, presented as mean with 95% confidence intervals. The Student’s t-test was used to compare the mean between two groups (survived and dead) for normally distributed continuous variables.

The biserial correlation coefficient was used to measure the degree of association between the outcome variable and continuous variables with a significance level of 0.05.

Categorical variables were presented as a proportion with 95% confidence intervals. The Chi-square test was performed to test the independence of attributes. Fisher’s Exact test was used whenever cell frequencies were less than five.

Crude odds ratios were computed for measuring the effect of each categorical covariates with outcome variables, i.e., death or survived. Logistic regression models were performed to predict mortality of COVID-19 taking all the significant covariates as independent variables.

In this study, four models were used to predict death using demographic variables like age and gender, signs and symptoms, severity of disease, comorbidities, and lab variables of COVID-19 cases after checking for the co-linearity of independent variables. Hosmer-Lemeshow test statistic was used to assess the goodness of fit for each model. Deviance test statistic was used to measure the discrepancy between the reduced model and the full model.

## Results

Table 1 presents the demographic and clinical features of the total of 984 COVID-19 cases admitted to our hospital during the study period. Out of 984 cases, 762 (77.44%) were males and 222 (22.56%) females. The overall case-fatality rate among admitted cases was 254 (25.81%) [males (26.64%) and females (22.96%)]. Differences in case-fatality rates due to gender was not found to be significant (crude odds ratio [95% CI] = 1.21 [0.856 - 1.73], p=0.272).

**Table 1:** Demographic distribution of COVID-19 cases in relation to mortality NIV: non-invasive ventilation

Variables	Dead (n=254)	Survived (n=730)	Unadjusted OR (95% CI)	Test Statistic	p-value
Male	203 (79.99)	559 (76.57)	1.22 (0.856 – 1.73)	Z=1.10	0.272
Age (Years) Mean (SD)	58.66 (14.82)	47.97 (16.16)	1.046 (1.035 – 1.057)	t-stat=9.26	0.0001
0 – 15	3 (1.18)	14 (1.91)	0.61 (0.174 – 2.14)	0.77	0.442
16 – 30	6 (2.36)	100 (13.69)	0.15(0.06 – 0.35)	4.41	0.0001
31 – 45	31 (12.20)	165 (22.60)	0.46 (0.314 – 0.72)	3.52	0.0001
46 – 60	80 (31.49)	263 (36.02)	0.816 (0.60 – 1.10)	1.30	0.192
61 – 75	99 (38.97)	165 (22.60)	2.19 (1.61 – 2.97)	5.01	0.0001
75+	35 (13.77)	23(3.15)	4.91 (2.84 -8.49)	5.07	0.0001
Severity					
Mild	35 (13.77)	439 (60.13)	0.61 (0.43 – 0.854)	-12.73	0.00001
Moderate	53 (20.86)	221 (30.27)	0.61 (0.43 – 0.854)	-2.86	0.004
Severe	166 (65.35)	70 (9.58)	17.84 (12.44 – 25.41)	15.80	0.0001
Admission in ICU	230 (90.55)	70 (9.58)	90.35 (55.50 – 147.09)	18.11	0.0001
Mean Duration of Hospital Stay	7.88 (6.11)	10.60 (5.71)	0.90 (0.87 – 0.93)	-6.17	0.0001
Oxygen Required	222 (87.40)	302 (41.36)	9.83 (6.59 – 14.65)	11.23	0.0001
NIV	94 (37.0)	19 (2.60)	21.98 (13.04 – 37.05)	11.60	0.0001
Invasive Ventilator	73 (28.74)	3 (0.04)	97.73 (30.44 – 313.65)	7.70	0.0001

The age of patients who succumbed to this disease was significantly higher at 58.66±14.82 years compared to those who survived 47.97±16.16 years (p=0.0001). Six dummy variables of 0-15 yrs, 16-30 yrs, 31-45 yrs, 46-60 yrs, 61-75 yrs, and 75+ were created for age groupings, and an increasing trend of fatality was observed as the age advances, with the highest risk of death among cases more than 75 years of age followed by age group 60-75 years.

Cases were categorized as mild, moderate, and severe based on the clinical features and presentation at the time of admission. Out of 984 cases, 476 (48.37%) were mild, 274 (27.84%) were moderate, and 136 (13.82%) were severe. Risk of mortality was found to be significantly higher (crude odds ratio = 17.84 [12.44 - 25.41]; p=0.0001) among severe cases as compared to mild and moderate cases.

Among 300 ICU admitted patients, 113 (37.66%) were managed with non-invasive ventilation (NIV). Risk of death among them was significantly higher (crude odds ratio = 21.98 [13.04 - 37.05]; p=0.0001). Almost one-fourth (25.33%) of ICU patients required invasive ventilation and these had a significant risk of dying (crude odds ratio = 97.73 [30.44 - 313.65]; p=0.0001).

We performed a binary logistic regression model taking death as a dependent variable and all covariates, which were found significant in bivariate analyses as independent variables as presented in Table 2. All the predictors as presented in Table 1 (Model 1) were found to be significantly associated with a high risk of mortality after controlling for the confounders.

**Table 2:** model-1: logistic regression model to predict mortality of COVID-19 with clinical conditions and demographic variables NIV: non-invasive ventilation

Covariates	Adjusted Odds Ratio with 95% CI	Z-statistic	P-value
Age 60-75 years	1.57 (0.89 – 2.74)	1.87	0.071
Age 75+ Years	3.42 (1.18 – 9.93)	2.26	0.024
Severe Cases	14.82 (7.85 – 27.97)	8.32	0.0001
Moderate Cases	2.55 (1.35 – 4.80)	2.90	0.004
ICU	44.94 (25.65 – 78.74)	13.30	0.0001
NIV	3.36 (1.69 – 6.69)	3.47	0.001
Constant	0.010 (0.0055 – 0.0194)	-14.32	0.0001

**Figure 1:**
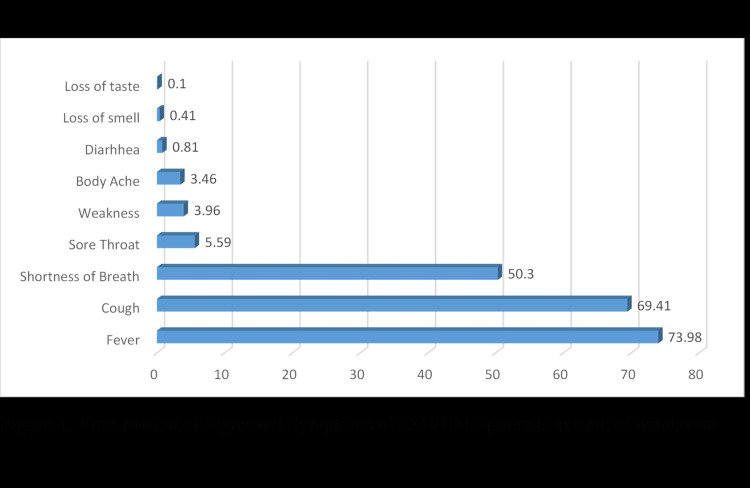
Presentation of signs and symptoms of COVID-19 at the time of admission

**Figure 2:**
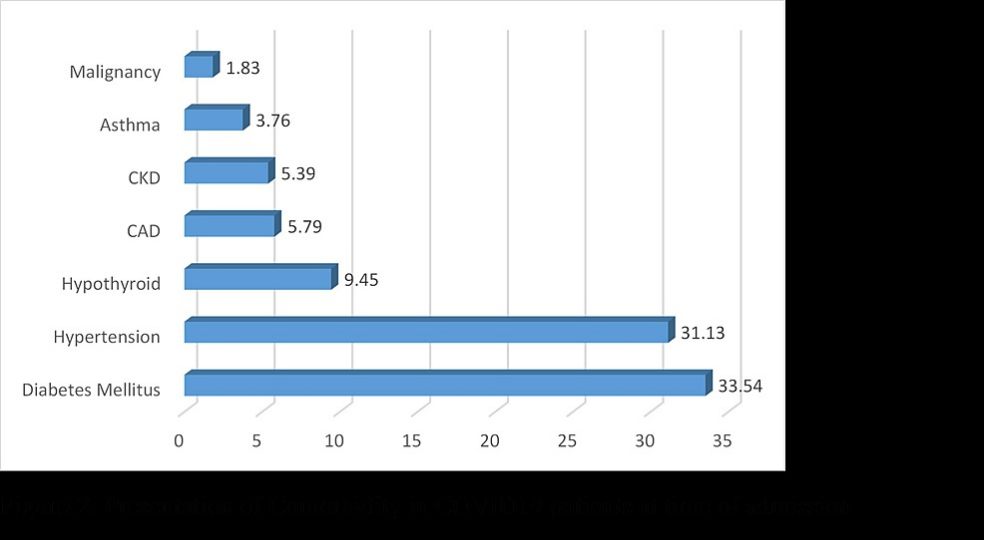
Presentation of comorbidity in COVID-19 patients at time of admission CKD: chronic kidney disease; CAD: coronary artery disease

The details of clinical symptoms and comorbidities are summarized in Table 3 and Figures 1, 2. The most common symptoms were fever (73.98%), cough (69.41%), and shortness of breath (50.3%)

**Table 3:** Comparison of clinical symptoms and comorbidities in relation to COVID-19 mortality CKD: chronic kidney disease; CAD: coronary artery disease

Variables	Death (n=254)	Survived (n=730)	Total (n= 984)	Test Statistic	p-value
Cough	177 (69.68)	22 (3.01)	199 (20.22)	Chi-square at 1d.f. = 22.78	0.0001
Shortness of Breath	185 (72.83)	310 (42.46)	495 (50.30)	Chi-square at 1d.f. = 69.52	0.0001
Fever >98.5	169 (66.53)	559 (76.57)	728 (73.98)	Chi-square at 1d.f. = 9.87	0.002
Weakness	17 (6.69)	22 (3.01)	39 (3.96)	Chi-square at 1d.f. = 6.70	0.068
Sore Throat	6 (2.36)	49 (6.71)	55 (5.58)	Chi-square at 1d.f. = 6.75	0.009
Diarrhea	1 (0.3)	7 (0.9)	08 (0.8)	Chi-square at 1d.f. = 0.746	0.388
Diabetic Mellitus	121 (47.63)	209 (28.63)	330 (33.53)	Chi-square at 1d.f. = 30.54	0.0001
Hypertension	122 (48.03)	184 (25.20)	306 (31.09)	Chi-square at 1d.f. = 45.64	0.0001
CKD	31 (12.20)	22 (3.01)	53 (5.38)	Chi-square at 1d.f. = 31.23	0.0001
CAD	21 (8.26)	36 (4.93)	57 (5.79)	Chi-square at 1d.f. = 3.84	0.050
Asthma	16 (6.29)	21 (2.87)	37 (3.76)	Chi-square at 1d.f. = 6.099	0.014
Hypothyroid	26 (10.23)	67 (9.17)	93 (9.84)	Chi-square at 1d.f. = 0.2465	0.620
Malignancy	8 (3.14)	10 (1.36)	18 (1.82)	Chi-square at 1d.f. = 3.324	0.068

The most common comorbidities reported were diabetes mellitus 330 (33.53%) cases followed by hypertension in 306 (31.09%), hypothyroidism 93 (9.45%), coronary artery disease (CAD) 57 (5.79%), chronic kidney diseases(CKD) 53 (5.38%), asthma 37 (3.76%) and different cancers 18 (1.83%). 

Bivariate analyses were performed to assess the effect of signs and symptoms, and comorbidities on mortality in the COVID-19 cases. The odds of mortality were 3.63 times more among cases having symptoms of shortness of breath than those cases without it (crude odds ratio = 3.63, 95% CI [2.65 - 4.96], p=0.0001).

Regarding the effect of co-morbidities

- Diabetes mellitus cases had higher odds of death (crude OR = 2.267, 95% CI [1.69 - 3.04], p=0.0001) compared to those without it.

- Cases with a history of hypertension were found to have a higher risk of death (crude OR = 2.737, 95% CI [2.03 - 3.68], p=0.0001) compared to those without any history of hypertension.

- Chronic kidney disease was also contributory to a higher risk of death (crude OR = 4.47, 95% CI [2.54 - 7.88], p=0.0001) compared to those without renal problems.

- Cardiovascular diseases were found to be associated with odds of death among COVID-19 cases but statistically not significant (crude OR = 1.737, 95% CI [0.994 - 3.03], p=0.052)

- Asthmatic patients were found to be at a higher risk of death compared to those without it (crude OR = 2.269, 95% CI [1.165 - 4.421], p=0.016).

- Co-existing malignancy with COVID-19 was found to be associated with a high risk of death (crude OR = 2.34, 95% CI: 0.913 - 5.99, p=0.072).

Binary logistic regression analysis was performed to model the risk of death among COVID-19 cases in the presence of various factors which were found to be significant in bivariate analysis. This was considered as Model 2 - taking signs and symptoms, and comorbidities as independent variables having p-values less than 0.10 in bivariate analysis. The result of the logistic regression model is presented in Table 4 (Model 2). All the predictors as presented in Table 4 were found to have a significant association with mortality after controlling for the confounders.

**Table 4:** Model 2 - logistic regression model to predict mortality in COVID-19 with signs and symptoms, and comorbidities CKD: chronic kidney disease

Covariates	Adjusted Odds Ratio with 95% CI	Z-statistic	P-value
Shortness of Breath	3.72 (2.66 – 5.19)	7.73	0.0001
Weakness	3.29 (1.59 – 6.79)	3.23	0.001
Diabetes Mellitus	1.51 (1.08 – 2.10)	2.42	0.016
Hypertension	2.20 (1.57 - 3.08)	4.59	0.0001
CKD	3.08 (1.66 – 5.735)	3.56	0.001
Malignancy	5.15 (1.84 – 14.41)	3.12	0.002

Based on the outcomes of Model 1 and Model 2, a final model (Model 3) was created to predict the risk of death taking all the demographic, clinical, signs and symptoms and comorbidity factors that were found significant in the bivariate analysis. Initially, all the covariates were included in the model, and the step-wise approach was used to drop the variables found insignificant in subsequent iterations. Collinear variables like admission in ICU, use of NIV were dropped from the model because of high association with severity status. The final model included the predictor variables for mortality as presented in Table 5.

**Table 5:** Model 3 - logistic regression model to predict mortality of COVID-19 with demographic, clinical, signs and symptoms, and comorbidities CKD: chronic kidney disease

Covariates	Adjusted Odds Ratio with 95% CI	Z-statistic	P-value
Age 60-75 years	1.56 (1.05 – 2.34)	2.19	0.029
Age 75+ Years	3.97 (1.94 – 8.15)	3.77	0.0001
Severe Cases	29.43 (17.35 – 49.93)	12.54	0.0001
Moderate Cases	1.96 (1.20 – 3.19)	2.71	0.007
Cough	3.19 (1.97 – 5.16)	4.73	0.0001
Shortness of Breath	2.13 (1.43 – 3.15)	3.75	0.0001
CKD	3.23 (1.56 – 6.67)	3.16	0.002

Laboratory parameters were also analyzed and a comparison was made between these values in COVID-19 patients who survived versus those who succumbed. Shapiro-Wilk test revealed none of the variables to be normally distributed (p<0.05), hence, a non-parametric test was applied to test the equality of median using the Mann-Whitney test (Table 6).

**Table 6:** Comparison of Laboratory Variables of COVID-19 cases at baseline in relation to mortality Hb: hemoglobin; SGPT: serum glutamic pyruvic transaminase; SGOT: serum glutamic oxaloacetic transaminase; ALP: alkaline phosphatase; STB: serum total bilirubin; ESR: erythrocyte sedimentation rate; CRP: C-reactive protein; LDH: lactate dehydrogenase; PT: prothrombin time; APTT: activated partial thromboplastin time; INR: international normalized ratio; RSB: random blood sugar

Variables	Death (n=254) Median (IQR)	Survived (n=754) Median (IQR)	Mann-Whitney Test Statistic (Z)	p-value
Hb (gm/dl)	11 (9.6 – 12.6)	12 (10.8 – 13.2)	5.780	0.0001
Total leukocyte Count (/mm3)	12.34 (8.0 – 18)	7.67 (6.0 – 10.6)	10.48	0.0001
Platelet Count (/mm3)	178.5 (116 – 281)	183 (133 -244)	0.236	0.8134
Neutrophil (%)	89.8 (81 – 93)	75.6 (65.4 – 85.7)	12.39	0.0001
Lymphocyte (%)	6.7 (4.3 – 13.7)	19.8 (11.0 – 28)	13.35	0.0001
Eosinophil (%)	0.70(0.20 – 1.3) (n=82)	0.90 (0.20 – 2.2) (n-489)	1.43	0.1527
SGPT (U/L)	57.2 (35.8 – 100.3) (n=253)	54.6 (34 – 96.6) (n=727)	0.84	0.4013
SGOT (U/L)	60.8 (39.7 – 104.9)	46.9 (33.5 – 75.5)	5.11	0.0001
ALP (U/L)	106 (81 – 144)	91.4 (73.7 – 116.6)	5.10	0.0001
STB (mg/dl)	1 (0.75 – 1.36) (n-248)	0.92 (0.71 – 1.13) (n=727)	3.16	0.0016
SDB (mg/dl)	0.36 (0.27 – 0.525) (n=244)	0.31 (0.24 – 0.40)	4.66	0.0001
Total Protein (g/dl)	6.45 (5.88 – 7.0)	7.2 (6.67 – 7.65)	11.23	0.0001
Albumin (g/dl)	3.20 (2.88 – 3.57)	3.825 (3.45 – 4.13)	14.07	0.0001
Globulin (g/dl)	3.21 (2.85 – 3.645)	3.34 (3.04 – 3.69)	3.94	0.0001
Urea (mg/dl)	57 (36.3 – 104)	28.7 (21.9 – 39.95)	13.57	0.0001
Creatinine (mg/dl)	1.0 (0.74 – 1.765)	0.76 (0.62 – 0.91)	8.49	0.0001
Sodium (mEq/L)	136 (131.7 – 140)	136 (133 – 138)	1.04	0.2972
Potassium (mEq/L)	4.42 (4.0 – 5.0)	4.3 (3.98 – 4.66)	1.91	0.0559
Calcium (mg/dl)	8.29 (7.9 – 8.83)	8.87 (8.5 – 9.37)	11.11	0.0001
ESR	65.5 (55 – 85) (n=46)	64 (39.5 – 82) (n=48)	1.07	0.2844
CRP (mg/L)	130.9 (49.75 – 207.38)	35.5 (6.01 – 102.33) (n=351)	8.24	0.0001
Ferritin (ng/ml)	735.7 (380.12 – 1410) (n=188)	339.55 (126.47 – 618.52) (n=498)	9.43	0.0001
D-Dimer (ng/ml)	1.93 (1.0 – 3.97) (n=183)	0.56 (0.38 – 1.23) (n=478)	10.83	0.0001
LDH (U/L)	1224 (789.3 – 1572.4) (n=137)	687.4 (529.33 – 967.07) (n=271)	8.84	0.0001
IL-6	15.01 (5.56 – 108.08) (n=4)	9.4 (2.91 – 51.5) (n=8)	0.68	0.4969
PT	14.0 (12.24 – 16) (n=210)	12.87 (12.06 – 14) (n=491)	5.52	0.0001
APTT	30.0 (26.0 – 37.0) (n=129)	31.15 (26.47 – 35.13) (n=345)	0.24	0.8095
INR	(0.99 – 1.133) (n=213)	0.96 (0.90 – 1.05) (n=501)	6.26	0.0001
RBS (mg/dl)	154.5 (122 – 224) (n=134)	136 (108 – 212) (n=174)	2.32	0.0201

Comparing the lab reports of patients who died compared to those who survived the disease, hemoglobin levels were significantly lower (11gm/dl vs 12gm/dl gm%, p=0.001), total leukocyte count on admission significantly raised (12.34/mm3 vs 7.37/mm3, p=0.0001), neutrophil counts significantly higher (89.8% vs 75.6%, p=0.0001), lymphocyte counts significantly lower (6.7% vs 19.8% p=0.0001). Kidney function tests like blood urea (57mg/dl vs 28.7mg/dl) and serum creatinine (1mg/dl vs 0.76mg/dl) were significantly higher (p=0.0001) whereas serum calcium was significantly lower (p=0.0001) among the cases who died (8.29mg/dl vs 8.87mg/dl). Biochemical markers such as CRP, ferritin, D-dimer, and lactate dehydrogenase (LDH) were significantly higher among the cases who died (p=0.0001). Prothrombin time and INR were significantly higher (p=0.0001) among the cases who died.

Lab variables were further categorized as normal and abnormal as categorical variables and compared between survivor and non-survivor (Table 7). Neutrophilia (>80% neutrophil), lymphopenia (<20% lymphocyte), hypoproteinemia (<6.4 gm/dl protein), and hypoalbunemia (<3.4 gm/dl albumin) were significantly more common in the non-survivors. Similarly, alanine transaminase (ALT), blood urea nitrogen and inflammatory markers like serum ferritin, LDH, and CRP were significantly raised in non-survivors. 

**Table 7:** Comparison of categorical laboratory variables of COVID-19 cases at baseline in relation to mortality Hb: hemoglobin; SGPT: serum glutamic pyruvic transaminase; SGOT: serum glutamic oxaloacetic transaminase; ALP: alkaline phosphatase; STB: serum total bilirubin; ESR: erythrocyte sedimentation rate; CRP: C-reactive protein; LDH: lactate dehydrogenase; PT: prothrombin time; APTT: activated partial thromboplastin time; INR: international normalized rate; RSB: random blood sugar; SDB: serum direct bilirubin

Variables	Death (n=254)	Survived (n=730)	Total (n-984)	Chi-square	p-value
Hb (g/dl) < 13	195 (76.77)	513 (75.71)	708 (71.95)	3.29	0.073
13-16	59	211	270		
Total Count (/mm3) >10 thousand	156 (61.11)	208 (28.48)	364 (36.99)	91.42	0.0001
4-10 thousand	94	521	615		
Platelet Count (/mm3) <150	97 (38.11)	247 (33.83)	344 (34.95)	1.57	0.222
150-450	157	783	640		
Neutrophil (%) >80	194 (77.95)	286 (33.17)	480 (48.78)	105.75	0.0001
40-80	59	444	503		
Lymphocyte (%) <20	218 (85.82)	371 (50.82)	589 (59.85)	95.72	0.0001
20-40	36	358	394		
NLR >3.5	221 (87.0)	392 (53.69)	613 (62.29)	90.28	0.0001
0.7 – 3.5	32	337	369		
Eosinophil (%) <1	48 (18.8)	251 (34.38)	299 (30.38)	1.46	0.235
1-6	34	238	272		
SGPT >40	172 (67.71)	475 (65.06)	647 (65.75)	0.5863	0.488
13 - 40	81	252	333		
SGOT >37	200 (78.87)	478 (65.47)	678 (68.90)	15.97	0.0001
0-37	53	251	304		
ALP >90	168 (66.14)	374 (51.23)	542 (55.08)	17.48	0.0001
30-90	85	356	441		
STB >1.2	81 (31.88)	141 (19.72)	222 (22.56)	18.51	0.0001
0.3 – 1.2	167	586	753		
SDB >0.2	222 (87.40)	637 (87.26)	859 (87.29)	2.29	0.137
0 – 0.2	22	92	114		
Total Protein <6.4	118 (46.45)	110 (15.06)	228 (23.27)	105.12	0.0001
6.4 – 8.3	135	620	755		
Albumin <3.4	163 (64.17)	150 (20.54)	313 (31.80)	166.68	0.0001
3.4 – 4.8	90	580	670		
Globulin >3.5	79 (31.10)	274 (37.53)	353 (35.87)	3.16	0.080
2 – 3.5	173	455	628		
Urea >43	168 (66.14)	142 (19.45)	310 (31.50)	188.16	0.0001
13-43	86	588	674		
Creatinine >1.3	78 (30.70)	53 (20.86)	131 (13.31)	95.70	0.0001
0.7-1.3	168	586	842		
Sodium >145	27 (10.62)	7 (0.9)	34 (3.45)	52.91	0.0001
135-145	226	127	947		
Potassium >5	51 (20.07)	95 (13.01)	146 (14.83)	7.53	0.008
3.5-5	202	634	836		
Calcium >10	4 (1.57)	29 (3.97)	33 (3.35)	3.34	0.071
8.6-10	249	698	947		
CRP >5	154 (60.62)	273 (37.39)	427 (43.33)	23.73	0.0001
0-5	08	78	86		
Ferritin >322	157 (61.81)	257 (35.20)	414 (42.07)	58.05	0.0001
24-322	31	241	272		
D-Dimer >0.5	172 (67.71)	277 (37.94)	449 (45.63)	78.89	0.0001
≤0.5	11	201	212		
LDH >460	133 (52.36)	222 (30.41)	355 (36.07)	0.0001	0.0001
230-460	04	49	53		
PT >16	49 (19.29)	32 (4.38)	81 (8.23)	40.69	0.0001
11-16	161	459	620		
APTT >34	46 (18.11)	105 (14.38)	151 (15.34)	1.18	0.319
24.8-34	83	240	323		
INR >1	89 (35.03)	183 (25.06)	272 (27.64)	1.75	0.207
0.8-1	124	318	442		

Logistic regression performed for significant variables found in the univariate analysis showed leukocytosis, high neutrophil-lymphocyte ratio (NLR), low albumin, high ferritin and D-dimer associated higher odds of death (Table 8).

**Table 8:** Logistic regression for Lab variable found significant in multivariate analysis

Variable	Adjusted Odd ratio	Z value	P value	95% Confidence interval
Total cell count (>10,000/mm3	1.749103	2.97	0.003	1.209812- 2.528789
Neutrophil-lymphocyte ratio (>3.3)	2.376507	3.62	0.000	1.486522- 3.79933
Serum total Bilirubin (STB) (>1.2 md/dl)	1.744037	2.78	0.005	1.178243- 2.581525
Serum total protein (<6.4 gm/dl)	2.101305	3.40	0.001	1.3695-3.223944
Serum Albumin (<3.4 g/dl)	2.302048	3.88	0.000	1.511396-3.506309
Blood Urea (>43 gm/dl)	3.722231	6.98	0.000	2.573338-5.38406
Ferritin (>322)	2.397764	2.53	0.012	1.21661- 4.725647
D-dimer (>0.5)	5.580757	3.65	0.000	2.216488- 14.05144

The final logistic regression model (Model 4) is presented in Table 9, taking variables of Model 3 and lab variable to predict death.

**Table 9:** Model 4 - final regression model to predict mortality of COVID-19 with demographic, clinical signs and symptoms, comorbidities, and lab variables

Covariates	Adjusted Odds Ratio with 95% CI	Z-statistic	P-value
Clinico-demographic variable			
Age 60-75 years	1.47 (1.02 – 2.21)	1.99	0.032
Age 75+ Years	3.97 (2.11 – 9.03)	3.97	0.0001
Severe disease	17.81 (11.14 – 28.47)	12.03	0.0001
Cough	3.83 (2.33 – 6.30)	5.29	0.0001
Shortness of Breath	2.35 (1.58 – 3.50)	4.24	0.0001
Chronic kidney disease	2.95 (1.40 – 6.21)	2.86	0.004
Lab variable			
Total cell count (>10,000/mm3	1.74 (1.20- 2.52)	2.97	0.003
Neutrophil-lymphocyte ratio (>3.3)	2.37 (1.48- 3.79)	3.62	0.000
Serum total protein (<6.4 gm/dl)	2.10 (1.36-3.22)	3.40	0.001
Serum Albumin (<3.4 g/dl)	2.30 (1.51-3.50)	3.88	0.000
Blood Urea (>43 gm/dl)	3.72 (2.57-5.38)	6.98	0.000
Ferritin (>322)	2.39 (1.21- 4.72)	2.53	0.012
D-dimer (>0.5)	5.58 (2.21- 14.05)	3.65	0.000

## Discussion

This retrospective observational study included analysis of 984 patients hospitalized with COVID-19 infection to identify the clinical and laboratory factors associated with mortality. The overall case-fatality rate (CFR) among the admitted cases was 25.81%. It varies across studies from less than 1 percentage to 18 percentage in Italy [4]. One of the largest cohorts of 5700 patients showed a case fatality of 21% [6]. In another study of 1697 patients, the CFR was 29.7% [7].

Our case-fatality rate was higher compared to some other reports [4,6,7]. This could be due to many socio-cultural-economic factors which play a role. Ours was a tertiary care referral hospital where only the sickest patients not admitted elsewhere were catered to. 

Discussing some factors individually

Gender: In these parts of the world, gender discrimination is prevalent. There are some studies that do report that the male gender has a higher risk for mortality [8-9]. Despite catering largely to a patriarchal society, in our study, no sex-based differences in mortality were noted.

Age: We found age as the most important risk factor for mortality in COVID-19 infections. The mean age of deceased patients was 10 years more than those who survived (58.66 years vs 47.97 years). The highest risk of death was noted amongst cases more than 75 years of age, followed by patients in the age group 60-75 years. More than 60% (60.34%) of patients with age more than 75 years died. Advanced age as a risk factor for mortality has been reported by several studies [10-13]. Wu C and colleagues concluded that older age was associated with a greater risk of development of ARDS and death likely owing to less rigorous immune response. [14]

Symptoms: Dyspnea on presentation was found to be significantly associated with mortality. The odds of dying were 3.63 times more among cases having shortness of breath on admission (crude odds ratio = 3.63). Du RH et al [15] have documented the presence of dyspnea, fatigue to be associated with a higher risk of mortality in COVID-19. Our study supported by another Indian study showed the presence of dyspnea significantly increased the risk of mortality [12]. We also noted that patients presenting with generalized weakness had a higher chance of progressing to severe disease. These symptoms may thus warrant more vigilant monitoring. Presence of fever (crude OR = 0.608, p=0.002) and sore throat (crude OR = 0.336, p=0.013) were not found to be associated with higher risk for mortality.

Comorbidities such as diabetes mellitus (DM), hypertension, CKD, and malignancy were seen to be significantly associated with increased risk of mortality. Studies had found that patients with chronic comorbidities were associated with severe COVID-19 infection with higher mortality [16-17]. The worse outcome in diabetes could be due to impaired innate immunity [17] which is the first line of defense against SARS-CoV-2 chronic inflammation or increased coagulation activity among DM patients [18]. It has been shown that diabetes and hypertension treatments with angiotensin-converting enzyme inhibitors (ACE inhibitors) and angiotensin II type I receptor blockers (ARBs) increase angiotensin-converting enzyme 2 (ACE2) expression which consequently raises the risk of developing severe and fatal complications. Similar to our study, Chilimuri et al [19] also showed higher odds of death in presence of comorbidities such as diabetes, hypertension, chronic obstructive pulmonary disease (COPD), CKD. A meta-analysis by Singh and Misra [20] also observed a significant 1.5 to 3-fold increase in severity of COVID-19 in patients with hypertension or diabetes or cardiovascular disease (CVD) or COPD or CKD and/or cancer. In the final predictive model, after controlling all confounders, diabetes and hypertension were not found as independent risk factors for mortality. Jain et al. [12] also showed diabetes is not an independent risk factor for mortality which is supporting our model.

Among lab variables, total cell count, neutrophil, random blood sugar, blood urea, creatinine was significantly higher, while serum calcium was significantly lower among the cases who died compared to survived cases (p=0.0001). Patients with severe disease had only a mild increase in WBC count while patients who died had a more clinically significant increase in this parameter. Leukocytosis, neutrophilia, and lymphopenia have been a well-proven risk factors for severe disease and worse outcome [21].

Literature suggests that D-dimer, CRP, serum ferritin, and LDH levels are important indicators of the severity of COVID-19 infections and may predict mortality in these patients [19]. This study also found that CRP, ferritin, D-dimer, and LDH were significantly higher among the cases who died (p=0.0001). Increased levels of D-dimer reflect increased coagulation activity which could be attributed to an increase in systemic pro-inflammatory cytokine responses which further contribute to atherosclerosis, plaque rupture. These activities further contribute to local inflammation as well as induction of procoagulant factors and thus predispose patients to ischemia and thrombosis [22-23]. Raised CRP levels, however, reflect the level of inflammation and it may activate complement level and enhance phagocytosis. CRP levels are an important indicator of the severity of COVID-19 infections [24]. Vargas-Vargas and Cortés-Rojo suggested serum ferritin levels as a key mediator suggesting immune dysregulation. Cytokine storm associated with raised serum ferritin levels has been associated with disease severity and adverse outcomes [25].

This study has certain limitations. Firstly, this is a retrospective single-center study which may limit the generalizability of the findings. Recording patient data only at the time of hospital admission could be another limitation of the study.

## Conclusions

The overall case-fatality rate among hospitalized COVID-19 patients at our hospital was 25.81%. Age more than 60 years, presence of CKD, and severe COVID-19 infection were the independent risk factors for mortality. Diabetes and hypertension did not independently increase the risk of mortality. The presence of cough or breathlessness was associated with worse outcomes while fever was associated with lower risk. Elevated CRP, ferritin, D-dimer, and LDH were associated with worse outcomes in COVID-19 infection. So, every hospitalized patient should undergo a detailed evaluation for comorbidities, the severity of disease, and these tests at the time of admission.
